# Functional disability and death wishes in older Europeans: results from the EURODEP concerted action

**DOI:** 10.1007/s00127-014-0840-1

**Published:** 2014-02-20

**Authors:** Madeleine Mellqvist Fässberg, Svante Östling, Arjan W. Braam, Kristoffer Bäckman, John R. M. Copeland, Manfred Fichter, Sirkka-Liisa Kivelä, Brian A. Lawlor, Antonio Lobo, Halggrimur Magnússon, Martin J. Prince, Friedel M. Reischies, Cesare Turrina, Kenneth Wilson, Ingmar Skoog, Margda Waern

**Affiliations:** 1 Neuropsychiatric Epidemiology Unit, Department of Psychiatry and Neurochemistry, Institute of Neuroscience and Physiology, Sahlgrenska Academy, University of Gothenburg, Gothenburg, Sweden; 2 Department of Epidemiology and Biostatistics, VU University Medical Centre, EMGO Institute for Health and Care Research, Amsterdam, The Netherlands; 3 Department of Resident Training, Altrecht Mental Health Care, Utrecht, The Netherlands; 4 Section of Old Age Psychiatry, Department of Psychiatry, University of Liverpool, Liverpool, UK; 5 Department of Psychiatry, Ludwig-Maximilians-Universität, Munich, Germany; 6 Schoen Klinik Roseneck, Prien, Germany; 7 Department of Family Medicine, University of Turku, Turku, Finland; 8 Department of Psychiatry, St. James’ Hospital, Jonathan Swift Clinic, Dublin, Republic of Ireland; 9 Instituto de Investigación Sanitaria Aragón (IIS Aragón) and CIBERSAM, Universidad de Zaragoza, Hospital Clínico Universitario, Zaragoza, Spain; 10 Department of Geriatrics, Landspitali, University Hospital of Iceland, Reykjavík, Iceland; 11 Health Services and Population Research Department, King’s College London, Institute of Psychiatry, London, UK; 12 Arbeitsgruppe Neuropsychologie Und Experimentelle Psychopathologie, Charité, Universitätsmedizin Berlin, Berlin, Germany; 13 University Psychiatric Unit, Department of Mental Health, Brescia University School of Medicine, Brescia Spedali Civili, Brescia, Italy

**Keywords:** Death wishes, Functional disability, Chronic conditions, Depressive symptoms, Late life

## Abstract

**Purpose:**

Physical illness has been shown to be a risk factor for suicidal behaviour in older adults. The association between functional disability and suicidal behaviour in older adults is less clear. The aim of this study was to examine the relationship between functional disability and death wishes in late life.

**Methods:**

Data from 11 population studies on depression in persons aged 65 and above were pooled, yielding a total of 15,890 respondents. Level of functional disability was trichotomised (no, intermediate, high). A person was considered to have death wishes if the death wish/suicidal ideation item of the EURO-D scale was endorsed. Odds ratios for death wishes associated with functional disability were calculated in a multilevel logistic regression model.

**Results:**

In total, 5 % of the men and 7 % of the women reported death wishes. Both intermediate (OR 1.89, 95 % CI 1.42; 2.52) and high functional disability (OR 3.22, 95 % CI 2.34; 4.42) were associated with death wishes. No sex differences could be shown. Results remained after adding depressive symptoms to the model.

**Conclusions:**

Functional disability was independently associated with death wishes in older adults. Results can help inform clinicians who care for older persons with functional impairment.

**Electronic supplementary material:**

The online version of this article (doi:10.1007/s00127-014-0840-1) contains supplementary material, which is available to authorized users.

## Introduction

Death wishes are not uncommon among older adults. Prevalence estimates range from 4 to 12 % [1–4]. While death ideation might reflect normal psychological processes in ageing, it may also indicate risk of future suicidal behaviour. In a population-based sample of 85-year-olds, death ideation was associated with either current symptoms of mental distress or past history of active suicidal ideation [5]; both are markers of potential risk.

Older adults who commit suicide often seek their doctors shortly before their deaths. However, physical ailments are often the focus of the consultation and many fail to communicate their despair [6]. Several case–control studies have demonstrated that physical illness is associated with increased suicide risk [7–13]. Record linkage studies utilising hospital registers [14, 15] and prescription registers [16] lend further support to the association between physical illness and suicide. How is this association mediated? There may be a direct connection between physical illness and depression. However, such an association may also be mediated by functional disability. Inability to carry out daily activities may lead to loss of autonomy, isolation and depression.

### What is known about the relationship between functional disability and suicidal behaviour?

In a clinical study of depressed older adults, a past suicide attempt was more common among patients who had impaired instrumental activities of daily living [17]. Two of the above mentioned case–control studies on completed suicide addressed the issue of functional disability. Conwell and colleagues [7] utilised the Lawton Instrumental Activities of Daily Living (IADL) and found that low IADL increased suicide risk. The Swedish study by Waern and colleagues [12] showed that people aged 75 years and above who required help with cooking, cleaning and shopping had an increased suicide risk compared to those who could carry out these activities on their own.

It remains unclear whether functional disability is associated with suicidal ideation independently of depression. Thus far, results are inconclusive, with some cross-sectional studies showing an independent association [18, 19], and others not [20]. A large multicenter study may provide further knowledge.

The EURODEP concerted action is a collaboration among research groups involved in population-based studies on depression in late life [21, 22]. With a total study sample of over 22,000, EURODEP provides the power to examine factors related to depression in later life. Previously reported findings from EURODEP [23] suggested a dose–response relationship between disability and depressive symptoms that was consistent across Western Europe. Eleven centres also had information regarding death wishes for a total of 15,890 persons. The aim of the current study was to explore the association between functional disability and death wishes, and to determine whether such a proposed relationship would be independent of depressive symptoms.

## Methods

### Samples

Representative samples of older persons aged 65–104 years (*n* = 15,890) living in Amsterdam (*n* = 3,987), Berlin, (*n* = 488), Dublin (*n* = 1,012), Reykjavik (*n* = 772), Liverpool (*n* = 3,366), London (*n* = 637), Ähtäri (*n* = 1,035), Gothenburg (*n* = 447), Munich (*n* = 346), Verona (*n* = 202), Zaragoza (*n* = 3,598) took part in psychiatric examinations. Inclusion criteria varied somewhat among centres. Sampling was based either on municipality registers or on general practitioner registers. For further information concerning the interviews and participants, the reader is referred to the detailed accounts of Copeland [21], Prince [22] and Braam [23].

## Measures

### Dependent variable: death wishes

Several instruments were used to assess death wishes (Table 1). Eight of the participating centres used the Geriatric Mental State scale (GMS) [24] and one used the short version of the Comprehensive Assessment and Referral Evaluation (SHORT-CARE) [25]. The questions employed were identical in both instruments (Have you felt that life was not worth living? Have you ever felt that you would rather be dead? Have you ever felt you wanted to end it all? Have you ever thought of doing anything about it yourself?). One centre used the Paykel question [26] (Have you ever wished that you were dead—for example, that you would fall asleep and never wake up again?) and another used an item from the Zung Self-Rating Depression Scale (ZSDS) [27] (I feel that others would be better off if I were dead). A person was considered to have death wishes if the death wish/suicidal ideation item of the EURO-D scale was endorsed. As decision trees differentiating between suicidal ideation and attempts varied at different sites, a more detailed analysis of specific types of suicidal behaviour was not possible.

**Table 1:** Demographic characteristics of the eleven EURODEP centres with data on death wishes (*n* = 15,890)

Centre	Country	Instrument psychiatric symptoms	Functional disability	Reference for disability scale applied	*n* (%)	Mean age (range)	Female, *n* (%)	Married, *n* (%)	High education, *n* (%)	MMSE <24, *n* (%)
Amsterdam	Netherlands	GMS	Interview	Katz et al. [29]	3,987 (25)	74 (65–84)	2,488 (62)	1,939 (49)	1,042 (26)	327 (8)
Berlin	Germany	GMS	Interview	Katz et al. [29]	488 (3)	84 (70–103)	240 (49)	148 (30)	147 (31)	97 (20)
Dublin	Ireland	GMS	Interview	–	1,012 (6)	74 (64–98)	648 (64)	495 (49)	301 (30)	141 (14)
Reykjavik	Iceland	GMS	Interview	Katz et al. [29]	772 (5)	86 (83–89)	463 (60)	216 (28)	166 (21)	–
Liverpool	England	GMS	Interview	Katz et al. [29], Prince et al. [57]	3,366 (21)	79 (69–104)	1,751 (52)	1,256 (37)	729 (22)	355 (11)
London	England	SHORT-CARE	Interview	Katz et al. [29], Prince et al. [57]	637 (4)	75 (65–99)	383 (60)	235 (37)	121 (19)	141 (22)
Ähtäri	Finland	ZSDS	Self-report	Zung et al. [27]	1,035 (7)	73 (65–95)	635 (61)	503 (49)	75 (7)	62 (6)
Gothenburg	Sweden	CPRS	Proxy interview	Östling and Skoog [58]	447 (3)	85	313 (70)	100 (23)	110 (26)	99 (22)
Munich	Germany	GMS	Observed	Oswald and Fleischmann [59]	346 (2)	88 (85–99)	268 (78)	63 (18)	103 (30)	128 (39)
Verona	Italy	GMS	Observed	Belloc et al. [60]	202 (1)	74 (65–100)	125 (62)	106 (53)	47 (23)	43 (21)
Zaragoza	Spain	GMS		Katz et al. [29]	3,598 (23)	77 (65–102)	2,115 (59)	1,897 (53)	498 (14)	414 (14)
All centres					15 890 (100)	77 (65–104)	9,429 (59)	6,958 (44)	3,339 (21)	1,807 (11)

Numbers of persons in denominators vary due to missing observations

*GMS* geriatric mental state, *ZSDS* Zung Self-Rating Depression Scale, *CPRS* Comprehensive Psychological Rating Scale

### Explanatory variables

#### Demographic variables

Education was assessed in several different ways at participating centres. Therefore, a range of index scores with variables between 0 and 1 was computed for the purpose of the EURODEP study [28]. Marital status was categorised as “married” vs. “non-married”, the latter category including those who were never married, divorced/separated or widowed.

### Perceived loneliness

Perceived loneliness was assessed (yes/no) at ten of the participating centres. The specific questions employed are shown by centre in the ESM Appendix 1.

### Functional disability and chronic condition

Most centres rated functional disability in accordance with the Katz scale [29]. Activities of daily living (ADL) are used to measure the individual’s ability to carry out everyday activities such as bathing, dressing, toileting, transfer, continence and feeding. To harmonise the ratings, total scores of the ADL scales were trichotomised into “no,” “intermediate”, or “high” levels of disability at each centre [23]. “High” level of disability was defined as the scores which fell in the highest tertile. Data regarding specific types of chronic conditions were not available at all centres, and ten centres had data on number of chronic conditions. Numbers were categorised as none, “one” and “two or more” [23].

### EURO-D score

The EURO-D harmonised scale was constructed by expert opinion with respect to correspondence between items on different depression scales (GMS [24], SHORT-CARE [25], ZSDS [27] and the Comprehensive Psychopathological Rating Scale (CPRS) [30]), and wherever possible by probabilistic modelling for some scales [22]. The EURO-D scale includes 12 items (depressive affect, pessimism, death wishes (as defined above), guilt, sleep problems, lack of interest, irritability, appetite problems, fatigue, reduced concentration, lack of enjoyment and tearfulness). Each item receives a rating of 0 (not present) or 1 (present). EURO-D scores can thereby range from 0 to 12, with higher scores reflecting greater depression symptom burden. For the purpose of this study, we removed the death wishes item from the EURO-D scale, yielding a maximum score of 11.

### Cognition and dementia

The Mini Mental State Examination (MMSE) [31] was used to assess cognitive functioning and was available in nine centres. In this study, MMSE score was analysed as a continuous variable. The diagnosis of dementia was based on the AGECAT algorithm [32] in nine centres. This algorithm has previously been validated against dementia diagnosis according to clinicians and against DSM-III-R criteria with satisfactory results [33, 34]. In the Gothenburg sample the diagnosis of dementia was based on DSM-III-R criteria, using tests of short- and long-term memory, abstract thinking, aphasia, apraxia, and agnosia [35]. Dementia was diagnosed using the Wilson Mental Capacity Scale [36] in Äthäri.

### Statistical analyses

A generalised linear mixed model with a logistic link function was used to analyse the association between sex, age, education, marital status, perceived loneliness, functional disability, number of chronic diseases, MMSE and EURO-D (independent variables) and death wishes (the dependent variable). Age was added as a continuous variable, and results are reported as how a 10-year increase in age increases the prevalence of death wishes. Plausible interactions (sex and functional disability; sex and chronic condition; sex and marital status; functional disability and depression) were also added to the model and tested. As the interactions proved sensitive to the inclusion or exclusion of other independent variables, a more parsimonious model containing only main effects that proved to be stable was in the end chosen. Results are presented as odds ratios (OR) and 95 % confidence intervals (CI). Odds ratios are what is termed “subject specific” and should thus be interpreted as the effect that a predictor variable has on the odds of developing death wishes for any given centre. See Agresti [37] for a detailed explanation. Further, the median odds ratio (MOR) was calculated from the intercept variance. This measure can be understood as the effect that belonging to a certain centre will have on the odds of having death wishes. If two random persons from two different centres who share the same covariate values were to be picked, one would have higher odds of death wishes and the other lower odds. Using the person with the higher odds in the numerator and the person with lower odds in the denominator repeating this procedure for every possible combination of subjects-centres would result in a distribution of odds ratios. The MOR is the median of this distribution and can be compared to the fixed effect estimates, yielding a measure of the relative size of the unexplained variation in comparison to the effects that explanatory variables have. For a more detailed explanation, see for instance Merlo [38]. Models were estimated using proc Glimmix in SAS 9.3 (SAS Institute Inc.). Descriptive statistics were analysed in IBM SPSS Statistics, version 20 for Windows.

## Results

**Fig. 1:**
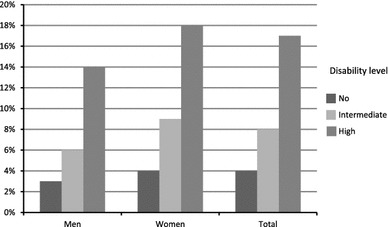
Prevalence of death wishes by disability level and sex within eleven EURODEP centres (*n* = 15,686)

Descriptive statistics are presented in Table 1. In total, 976 individuals (6 %) reported death wishes, with a similar rate among men and women (Table 2). Rates of death wishes ranged from 3 to 27 % with the lowest prevalence in Amsterdam and Zaragoza and the highest in Munich. Figure 1 shows the numbers and proportions of men and women with death wishes by disability level. A linear association between disability level and death wishes was observed, indicating a dose–response relationship in both men and women.

**Table 2:** Prevalence of death wishes by centre and sex

	Death wishes		
	Men, *n* (%)	Women, *n* (%)	Total, *n* (%)
Amsterdam	40 (3)	87 (3)	127 (3)
Berlin	51 (21)	67 (28)	118 (24)
Dublin	29 (8)	67 (10)	96 (9)
Reykjavik	7 (3)	29 (6)	36 (5)
Liverpool	66 (4)	88 (5)	154 (5)
London	23 (9)	40 (10)	63 (10)
Ähtäri	15 (4)	71 (11)	86 (8)
Gothenburg	12 (9)	47 (15)	59 (13)
Munich	24 (31)	69 (26)	93 (27)
Verona	3 (4)	22 (18)	25 (12)
Zaragoza	31 (2)	88 (4)	119 (3)
All centres	301 (5)	675 (7)	976 (6)

Results of the multivariate model showed a nearly twofold increase in odds of having death wishes among the group with an intermediate disability level; the increase was threefold among those with high disability level (Table 3). We found no association between a 10-year increase in age and death wishes. Being unmarried was not associated with death wishes. Perceived loneliness was strongly associated with death wishes, with the effect being nearly fourfold.

**Table 3:** Multivariate model showing odds ratios of having death wishes (*n* = 11,030)

Fixed (within centre) effects	OR (95 % CI)	p**
Functional disability (no.)	REF	<0.0001
Intermediate vs. (no.)	1.894 (1.420; 2.526)	
High vs. (no.)	3.220 (2.344; 4.422)	
Sex (female)	1.180 (0.967; 1.440)	0.1038
Age Δ = 10	0.875 (0.759; 1.009)	0.0664
Education index Δ = 0.1	1.017 (0.960; 1.077)	0.5627
Marital status (not married)	1.214 (0.928; 1.589)	0.1315
Perceived loneliness	3.975 (3.285; 4.809)	<0.0001
Chronic condition (0)	REF	0.0013
1 vs. (0)	1.324 (1.051; 1.669)	
2 or more vs. (0)	1.795 (1.374; 2.343)	
MMSE	1.050 (1.030; 1.071)	<0.0001

Random effects	Variance $\varvec{\sigma}^{2}$	MOR	*p* value
Centre	0.8673	2.4311	<.0001

Based on data from 8 centres. Reykjavik was excluded due to missing data on MMSE, Dublin due to missing data on Chronic condition and Verona due to missing data on Perceived loneliness

** Type 3 tests used

The effect of having death wishes was slightly increased by the presence of one chronic condition, and nearly twofold for two or more chronic conditions. Further, a one-point decrease in MMSE score increased the odds of having death wishes by 5 % (OR 1.050 (1.030; 1.071),* p* ≤ 0.0001). There was also a significant between-centre variation, as calculated by the MOR showing that the unexplained heterogeneity was higher than the effect of intermediate functional disability, although not as large as the effect of high functional disability.

To determine whether the relationship between functional disability and death wishes was independent of depressive symptoms, the Euro-D score was added to the multivariate model (Table 4). While the effect of the predictor variables decreased, all variables that were associated with death wishes in the initial model remained significant. The strongest effect of having death wishes was found for the group who reported loneliness. As individuals with dementia might have difficulties understanding the questions, we reanalyzed the multivariate model after excluding all individuals who fulfilled criteria for dementia (*n* = 734); this did not affect our results (ESM Appendix 2, Table 5).

**Table 4:** Multivariate model showing odds ratios of having death wishes with inclusion of depression (*n* = 11,030)

Fixed (within centre) effects	OR (95 % CI)	p**
Functional disability (no.)	REF	0.0002
Intermediate vs. (no.)	1.602 (1.196; 2.146)	
High vs. (no.)	2.439 (1.767; 3.366)	
Sex (female)	1.064 (0.869; 1.302)	0.5491
Age ∆ = 10	0.865 (0.748; 0.999)	0.0489
Education index ∆ = 0.1	1.027 (0.969; 1.088)	0.3685
Marital status (not married)	1.365 (1.041; 1.789)	0.0299
Perceived loneliness	2.720 (2.231; 3.317)	<0.0001
Chronic disease (0)	REF	0.0325
1 vs. (0)	1.210 (0.957; 1.530)	
2 or more vs. (0)	1.459 (1.110; 1.917)	
MMSE	1.033 (1.013; 1.055)	0.0013
Euro-D	1.783 (1.635; 1.945)	<0.0001

Random effects	Variance *σ* ^2^	MOR	*p* value
Centre	0.731	2.26044	<.0001

Based on data from 8 centres. Reykjavik was excluded due to missing data on MMSE, Dublin due to missing data on chronic condition and Verona due to missing data on Perceived loneliness

** Type 3 tests used

## Discussion

To our knowledge, this is the largest population-based study of functional disability and death wishes in older adults. Functional disability was associated with death wishes and this relationship remained also after adjusting for depressive symptom score. A dose–response relationship was observed in both men and women regarding death wishes and disability level.

We found that 6 % of Europeans over the age of 65 years reported death wishes. Our result that functional disability was independently associated with death wishes is in line with the previous studies of “younger” older [39, 40] and older adults [18, 19]. Having one or two or more chronic conditions was also associated with death wishes among men and women. Similar results were found in studies examining death wishes [3], suicide attempt [41] and completed suicide [16, 42]. One primary care based study from Australia showed that the number of chronic conditions was associated with suicidal thoughts in persons aged 60 years and above, but the association did not remain in a multivariate model that included a large number of clinical and sociodemographic characteristics [43].

We could not show a sex difference regarding the strength of the association between functional disability and death wishes, and this was the case for chronic conditions as well. The literature on this remains inconclusive. While there are several studies suggesting that physical illness and disability is more strongly related to both fatal and non-fatal suicidal behaviour in older men than in women [11, 15, 44], a recent study focusing on a somewhat younger age group (55–74 years) found no such association [45].

Perceived loneliness was strongly associated with death wishes. Similar results have been found in two recently published studies consisting of individuals aged 58–98 years [46] and in 65–75 year-olds [47]. Previous studies of older adults have also shown that feelings of loneliness were associated with both suicide attempts [48] and suicide [9]. Our results regarding loneliness indicate that individuals with limited social networks may be more vulnerable in the face of functional disability. Deteriorating functional ability might lead to persons becoming more isolated, and vice versa. A recently published study from Denmark showed that functional decline in men who live alone may be delayed in the presence of strong social relationships [49].

## Methodological considerations

Some limitations need to be addressed. Participation rates at the different centres varied but it is unclear how this might have affected results. Further, there are problems associated with the functional disability categories utilised in this study. Several different rating scales were used to measure functional disability. Disability categories are trichotomised at each site, which means that two individuals with identical levels of disability may end up in different disability categories. Data on specific types of functional disability are lacking. Also, while we have data on the number of chronic conditions, we have no information concerning the severity of these. Similarly, the Euro-D score measures numbers of symptoms but not severity of each item. Another limitation was the lack of IADL data which provide a measure on the level of independence. Previous research suggests that IADL is more strongly associated with suicidal ideation and suicide attempts than ADL [40, 45].

While the death wish item is available at all centres, it encompasses a large range of suicidal feelings; death wishes/suicidal thoughts. More precisely defined questions about suicidal ideation would be advantageous, but heterogeneous decision trees employed at the different sites make more specific grading of suicidal ideation a difficult and uncertain task.

We chose not to compare prevalence figures for death wishes in the participating centres. Even centres that employ exactly the same instrument (GMS) may obtain different information because interviewers may have emphasised certain words or been more exhaustive in their mode of questioning. Also, there may be cultural differences concerning the participants’ willingness to report thoughts about death and suicide to the interviewer. A different study set-up, employing collateral information sources might reveal higher rates of death wishes [6].

We do not have access to cause-of-death data. It can be assumed that a number of cases of completed suicide have occurred during the time that passed since the interviews. A prospective study, that also included death by suicide as an outcome would provide more information. Finally, the study design did not allow us to include other potentially relevant factors such as duration of illness/disability. While we were able to examine the association between loneliness and death wishes, other pertinent social support factors [50] could not be studied.

## Implications for suicide prevention

Functional disabilities are common among older persons, in particular among those who seek health care. Some may have a tendency to experience particular difficulties in coping with age-related physical impairment and other losses that may occur during later life. Persons with brittle thinking may be at particular risk for suicide [51] because they have a rigid view of themselves and their surroundings, lacking the capacity for adaptation to new experiences. Anankastic (obsessional) and anxious traits have also been linked with senior suicide [52]. It is important that physicians inquire about death wishes and suicidal feelings, especially when older patients indicate that they are having trouble coping with their functional disabilities. The detection of depressive symptoms is especially important in physically impaired seniors as functional impairments [53] and physical conditions [54] improve with treatment for depression. Gatekeeper referral programs have been shown to have a positive effect on the reduction of both social isolation and suicidal ideation in older adults [55]. Also, involvement of relatives/close friends in treatment planning could have a positive effect, as these persons can provide insight regarding the life situation and may also be more likely to detect early changes in symptomatology. Providing telephone support to somatically frail individuals via a TeleHelp-TeleCheck Service [56] may reduce social isolation, making it a promising strategy. Future research could involve testing of community interventions to engage older adults with physical and functional issues.

## Electronic supplementary material

Below is the link to the electronic supplementary material.

Supplementary material 1 (DOCX 12 kb)

Supplementary material 2 (DOCX 14 kb)
