# Supplementary material for: Functional disability and death wishes in older Europeans: results from the EURODEP concerted action
Source: Soc Psychiatry Psychiatr Epidemiol. 2014 Feb 20;49(9):1475–82. doi: 10.1007/s00127-014-0840-1 (PMC4143593; doi:10.1007/s00127-014-0840-1)
Supplement: Supplementary file 1 — Supplementary material 1 (DOCX 12 kb) [file 127_2014_840_MOESM1_ESM.docx]

**Appendix 1**

Amsterdam, Berlin, Dublin, Reykjavik, Liverpool, Munich, Zaragoza: Do you feel lonely? 0=No

1=Feels lonely

2=Feels very lonely

8=Does not know/No answer

9=Not asked

Coding: 0=0 (no), 1-2=1 (yes) (all else missing)

London: Have you felt lonely in the past month?

0=No

1=Yes

Athäri: How often do you feel lonely?

1. Often

2. Sometimes

3. Never

Coding: 3=0 (no), 1-2=1 (yes)

Gothenburg: Do you feel lonely? If so, how often?

1. Never

2. Rarely

3. Sometimes

4. Often

Coding: 1=0 (no), 2, 3, 4=1 (yes)
