# Supplementary material for: Functional disability and death wishes in older Europeans: results from the EURODEP concerted action
Source: Soc Psychiatry Psychiatr Epidemiol. 2014 Feb 20;49(9):1475–82. doi: 10.1007/s00127-014-0840-1 (PMC4143593; doi:10.1007/s00127-014-0840-1)
Supplement: Supplementary file 2 — Supplementary material 2 (DOCX 14 kb) [file 127_2014_840_MOESM2_ESM.docx]

**Appendix 2**

**Table 5** Multivariate model showing odds ratios of having death wishes with inclusion of depression

excluding individuals with dementia (n=10296*)

|  |  |  |  |
| --- | --- | --- | --- |
| Fixed (within centre) effects |  | OR (95 % CI) | P** |
| Functional disability (no)  Intermediate vs. (no)  High vs. (no)  Sex (female)  Age ∆=10  Education index ∆=0,1  Marital status (not married)  Perceived loneliness  Chronic condition (0)  1 vs. (0)  2 or more vs. (0)  MMSE  Euro-D |  | REF  1.524 (1.105; 2.101)  2.207 (1.540; 3.163)  1.115 (0.896; 1.388)  0.860 (0.734; 1.008)  1.018 (0.954; 1.086)  1.231 (0.923; 1.642)  2.879 (2.319; 3.574)  REF  1.131 (0.876; 1.461)  1.455 (1.080; 1.961)  1.069 (1.035; 1.062)  1.853 (1.688; 2.034) | 0.0013  0.3302  0.0622  0.5936  0.1321  <.0001  0.0504  <.0001  <.0001 |
|  |  |  |  |
| Random effects  Centre | Variance $\boldsymbol{\sigma}^{\boldsymbol{2}}$  0.8072 | MOR  2.35611 | p-value  <.0001 |
|  |  |  |  |

* Based on data from 8 centres. Reykjavik was excluded due to missing data on MMSE, Dublin due to

missing data on Chronic condition and Verona due to missing data on Perceived loneliness.

** Type 3 tests used
